# Canonical WNT/β-Catenin Signaling Activated by WNT9b and RSPO2 Cooperation Regulates Facial Morphogenesis in Mice

**DOI:** 10.3389/fcell.2020.00264

**Published:** 2020-05-08

**Authors:** Yong-Ri Jin, Xiang Hua Han, Katsuhiko Nishimori, Dan Ben-Avraham, Youn Jeong Oh, Jae-won Shim, Jeong Kyo Yoon

**Affiliations:** ^1^Center for Molecular Medicine, Maine Medical Center Research Institute, Scarborough, ME, United States; ^2^Department of Applied Biological Chemistry, Tohoku University, Sendai, Japan; ^3^Nancy and Stephen Grand Israel National Center for Personalized Medicine, Mantoux Institute for Bioinformatics, Weizmann Institute of Science, Rehovot, Israel; ^4^Soonchunhyang Institute of Medi-Bio Science, Soonchunhyang University, Cheonan-si, South Korea; ^5^Department of Integrated Biomedical Science, Soonchunhyang University, Cheonan-si, South Korea

**Keywords:** R-spondin2, Wnt9b, WNT signaling, facial development, cleft lip, cleft palate

## Abstract

The R-spondin (RSPO) family of proteins potentiate canonical WNT/β-catenin signaling and may provide a mechanism to fine-tune the strength of canonical WNT signaling. Although several *in vitro* studies have clearly demonstrated the potentiation of canonical WNT signaling by RSPOs, whether this potentiation actually occurs in normal development and tissue function *in vivo* still remains poorly understood. Here, we provide clear evidence of the potentiation of canonical WNT signaling by RSPO during mouse facial development by analyzing compound *Wnt9b* and *Rspo2* gene knockout mice and utilizing *ex vivo* facial explants. *Wnt9b;Rspo2* double mutant mice display facial defects and dysregulated gene expression pattern that are significantly more severe than and different from those of *Wnt9b* or *Rspo2* null mutant mice. Furthermore, we found suggestive evidence that the LGR4/5/6 family of the RSPO receptors may play less critical roles in WNT9b:RSPO2 cooperation. Our results suggest that RSPO-induced cooperation is a key mechanism for fine-tuning canonical WNT/β-catenin signaling in mouse facial development.

## Introduction

In mice, WNT/β-catenin signaling plays a significant role during facial development. Dysregulation of WNT/β-catenin signaling by ablation or activation of the *Ctnnb1* (β*-Catenin*) gene function within facial primordia results in severe defects in many facial structures (Brault et al., 2001; Reid et al., 2011; Wang et al., 2011; He and Chen, 2012). Similarly, mice lacking the *Lrp6* WNT receptor gene, or both the *Lrp5*, another WNT receptor gene, and *Lrp6* genes also exhibit severe facial developmental deficits (Song et al., 2009; Joeng et al., 2011), distinctly indicating the specific roles of WNT/β-catenin signaling in facial structure development. Multiple WNT ligands and their co-regulators are expressed within facial primordia in mouse embryos (Summerhurst et al., 2008; Geetha-Loganathan et al., 2009). Among them, *WNT3* and *WNT9B*/*Wnt9b* mutations are associated with cleft palate/lip phenotype in humans and mice, respectively (Niemann et al., 2004; Menezes et al., 2010; Jin et al., 2012; Fontoura et al., 2015), suggesting that they are specific WNT ligands critical for facial development. Intrinsic differences among WNT ligands and the presence of their extracellular coactivators and inhibitors can control the specificity and strength of WNT/β-catenin signaling. However, the mechanism by which WNT3 and WNT9b integrate with other WNT signaling regulators to generate fine-tuned WNT signaling during facial morphogenesis is still unclear.

The R-spondin (RSPO) family of proteins are known for their roles in potentiating or synergistically activating canonical WNT/β-catenin signaling in the presence of the WNT ligands (Jin and Yoon, 2012; Raslan and Yoon, 2019). RSPOs inhibit activities of plasma membrane-bound E3 ubiquitin ligases, zinc and ring finger 3 (ZNRF3), and ring finger 43 (RNF43), both of which are specifically engaged in the degradation of the WNT receptors, Frizzleds (FZDs) and likely LRP5/6 (Hao et al., 2012). RSPOs simultaneously bind ZNRF3/RNF43 and leucine-rich repeat-containing G protein-coupled receptor 4/5/6 (LGR4/5/6) to induce endocytosis of ZNRF3/RNF43 (Xie et al., 2013). Therefore, expression levels of WNT receptors on the plasma membrane increase, resulting in sensitization of the signaling response to the WNT ligands (Wang et al., 2011). Alternatively, independent from the ZNRF3/RNF43-mediated mechanism, RSPOs synergistically activate WNT/β-catenin signaling through LGR4 and the associated scaffold protein, IQ motif-containing GTPase-activating protein 1 (IQGAP1) (Carmon et al., 2014). Upon binding of RSPO to LGR4, IQGAP1 brings RSPO-LGR4 to the WNT signaling complex through enhanced IQGAP1-DVL interaction. As a scaffold, IQGAP1 binds a plethora of intracellular signaling molecules, including MAP kinases, and modulates their activities (Carmon et al., 2014). The interaction between IQGAP1 and MEK1/2 potentiates β-catenin-dependent signaling by promoting phosphorylation of WNT receptor LRP5/6 (Carmon et al., 2014). Furthermore, there is emerging evidence that supports LGR4/5/6-independent WNT signaling activation by the cooperative action of WNT and RSPO (Lebensohn and Rohatgi, 2018; Szenker-Ravi et al., 2018; Raslan and Yoon, 2019). Therefore, RSPOs play critical roles in regulating the activation of WNT/β-catenin signaling by different mechanisms. Despite an accumulation of data in recent years, there has been no confirmation as to whether RSPOs along with WNT ligands indeed potentiate or cooperatively activate WNT/β-catenin signaling *in vivo*, especially during development.

Previously, we reported that the inactivation of the *Rspo2* gene results in reduced WNT/β-catenin signaling mainly within the mandibular branchial arch 1 (MdBA1), resulting in cleft palate accompanying the deformation of MdBA1-derived bone structures (Jin et al., 2011). In this study, we proposed that unknown WNT ligands that are expressed in the ectoderm of MdBA1 are likely to cooperate with mesenchymal-derived RSPO2 to regulate MdBA1 morphogenesis and subsequently jawbone development. Mice lacking the *Wnt9b* gene exhibited cleft lip with cleft palate, which resulted from a retarded outgrowth and subsequent failed fusion of the nasal processes (NP) and maxillary process of branchial arch 1 (MxBA1) due to significantly diminished WNT/β-catenin signaling (Jin et al., 2012). Although the facial defects are mainly restricted to the upper jaw in *Wnt9b* mutant mice and the lower jaw in *Rspo2* mutant mice, respectively, considering the robust *Wnt9b* expression in facial processes, it is highly probable that WNT9b is a specific ectoderm-derived WNT ligand working cooperatively with mesenchyme-derived RSPO2 to regulate WNT/β-catenin signaling during facial development.

In the present study, we systematically investigated how *Wnt9b* and *Rspo2* genes function cooperatively to regulate WNT/β-catenin signaling during facial development by utilizing *in vivo* “loss of function” mouse models and *ex vivo* “gain of function” facial explants cultures. *Wnt9b;Rspo2* double-gene knockout (DKO) mice exhibited defective facial phenotypes that were much more severe than those of *Wnt9b* or *Rspo2* single-gene knockout mice. We found that WNT/β-catenin signaling within the facial primordia is activated by the cooperative action between ectoderm-derived WNT9b and mesenchyme-derived RSPO2 and is crucial for growth and patterning of the facial structures in mice. Intriguingly, our results suggest that the LGR4 family of RSPO receptors may play less significant roles in WNT9b:RSPO2-mediated WNT signaling regulation in the development of facial structures.

## Results

### RSPO2 and WNT9b Synergistically Induce WNT/β-Catenin Signaling

**FIGURE 1:**
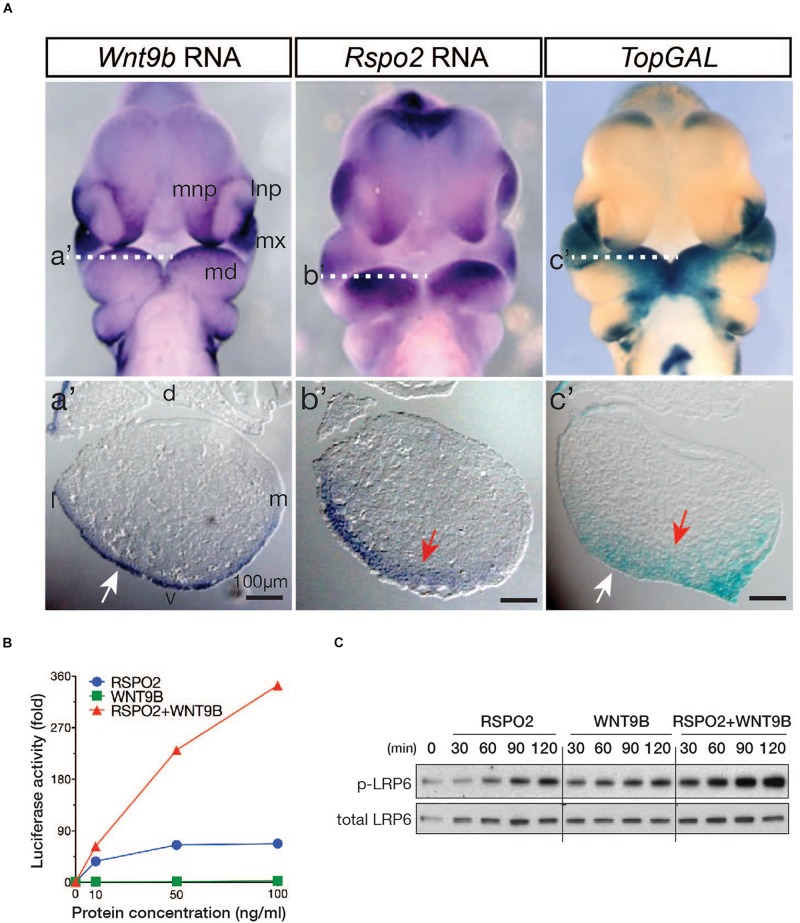
**(A)** Expression of the *Wnt9b* and *Rspo2* genes and a WNT reporter, *TopGAL*, in mouse embryos at E10.5. Frontal images are shown. Transverse section planes within the BA1 are marked by white-dotted lines, and the matching images of transverse sections are shown as a’, b’, and c’. Only the left side of the BA1 is shown. Orientation of the sections is indicated by dorsal/ventral (d/v) and lateral/medial (l/m) labels. **(B)** *STF* reporter assay. HEK293T cells were transfected with 20 ng of WNT signaling *STF* reporter and 10 ng TK-*Renilla* luciferase DNA in triplicate in 48-well plates. At 2 days after transfection, cells were treated with recombinant WNT9b and RSPO2 proteins with the indicated concentrations for 24 h. *STF* luciferase activities were normalized by *Renilla* luciferase activities. **(C)** Phosphorylated and total LRP6 protein levels were determined by Western blot analysis. HEK293T cells were treated with WNT9b (20 ng/ml) and RSPO2 (200 ng/ml) proteins for the indicated duration.

We have previously proposed that RSPO2 expressed within the ectomesenchyme of mandibular branchial arch1 (BA1) may potentiate canonical WNT/β-catenin signaling with unknown WNT ligand(s) expressed in ectodermal epithelial cells of BA1 (Jin et al., 2011). Considering the known facial defects observed in *Wnt9b* gene KO mice (Jin et al., 2011, 2012), we reasoned that WNT9b, among the WNT ligands expressed in the BA1, likely activates WNT/β-catenin signaling in a cooperative manner with RSPO2 within the BA1. We first compared *Wnt9b* and *Rspo2* expression in the facial processes of mouse embryos at E10.5 (embryonic 10.5 days post coitum). We detected *Wnt9b* gene expression within the ectoderm of both the BA1 and NPs adjacent to the *Rspo2* expression domain (Figure 1A). The expression of a WNT/β-catenin signaling reporter, *TopGAL* transgene, was detected within both the ectoderm and mesenchyme of the BA1 (Figure 1A). Their expression domains suggest that WNT9b is likely the previously suspected ectoderm-derived WNT ligand and that, together with RSPO2, it may regulate the activation of WNT/β-catenin within the facial processes including BA1.

To determine whether WNT9b and RSPO2 cooperatively induce WNT/β-catenin signaling, we examined the activity of WNT/β-catenin signaling reporter *SuperTopFlash* (*STF*) in HEK293T cells treated with recombinant RSPO2 and WNT9b proteins. While WNT9b or RSPO2 alone only induced a weak to mild activation of the *STF* reporter, combined treatment of RSPO2 and WNT9b produced a strong synergistic activation of the *STF* reporter in a dose-dependent manner (Figure 1B). Furthermore, phosphorylation of the LRP6 receptor at serine 1490, the earliest indication of WNT/β-catenin activation, was also synergistically enhanced within 30 min with co-treatment of the RSPO2 and WNT9b ligands (Figure 1C). These *in vitro* data raise the possibility that RSPO2 and WNT9b expressed within the BA1 and NPs may cooperate to activate WNT/β-catenin signaling.

**FIGURE 2:**
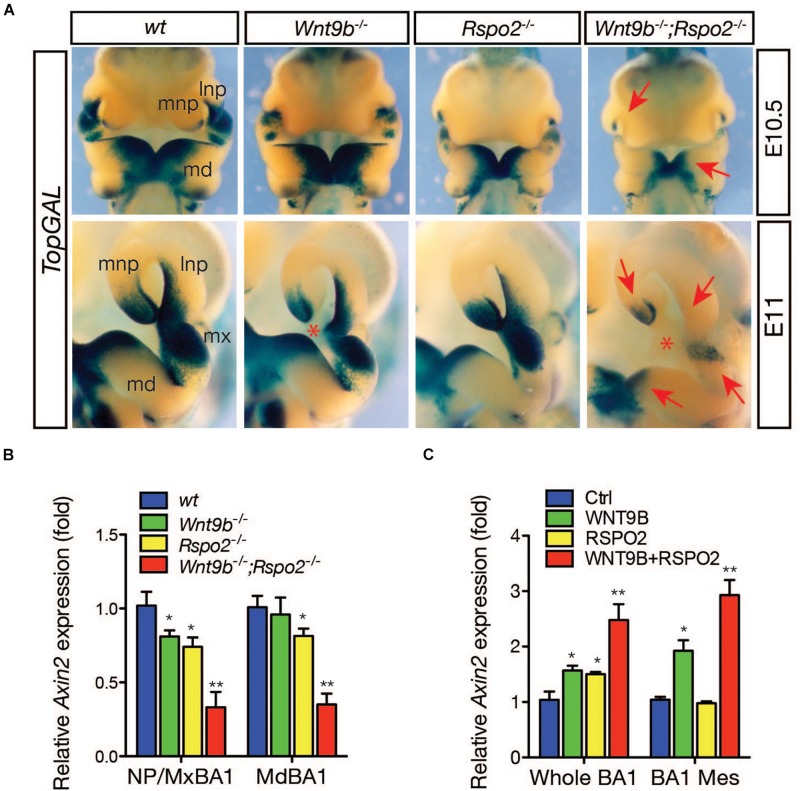
**(A)** Expression of WNT/β-catenin signaling reporter, *TopGAL* transgene, in the facial processes at E10.5-11 embryos (*n* = 3 for each embryonic age). To correctly visualize *TopGAL* expression, we used *Rspo2^Δ*ZN*^* null mice in which the *LacZ* and neomycin-resistance genes inserted into the *Rspo2* locus were removed. Embryos were incubated with X-gal substrate for 8 h. Red arrows indicate reduced or absent *TopGAL* expression. Red asterisk indicates a gap between lnp and mnp produced by a failure of nasal process fusion. lnp, lateral nasal process; mdBA1, mandibular process of the branchial arch 1; mnp, medial nasal process; mxBA1, maxillary process of the branchial arch 1. **(B)** qRT-PCR analysis for *Axin2* expression in the facial process explants (*n* = 4, NP/MxBA1, nasal process/maxillary branchial arch 1 and MdBA1, mandibular branchial arch 1) dissected from *Wnt9b*KO, *Rspo2*KO, and DKO embryos at E10.5. **(C)** qRT-PCR analysis for *Axin2* expression in the facial process explants (*n* = 4, whole BA1, whole mandibular branchial arch 1 and BA1 Mes, mesenchymal part of the mandibular branchial arch 1) cultured in the presence of WNT9b (20 ng/ml) and/or RSPO2 (200 ng/ml) proteins. Error bars represent the standard error of the mean (SEM). *p < 0.05; **p < 0.01.

To determine whether WNT9b and RSPO2 together can potentiate WNT/β-catenin signaling *in vivo*, we examined the expression of a WNT/β-catenin signaling reporter, *TopGAL* transgene, in *Wnt9b^–/–^*, *Rspo2^–/–^*, and *Wnt9b^–/–^;Rspo2^–/–^* (DKO) embryos at E10.5 and E11.0, respectively (Figure 2A). As previously reported (Jin et al., 2011, 2012), *TopGAL* expression was mainly diminished in the MNP of *Wnt9b^–/–^* embryos and the BA1 of *Rspo2^–/–^* embryos. However, the *TopGAL* expression domain was significantly reduced in both the BA1 and NPs of the DKO embryos, with a significantly lower expression level than those in *Wnt9b* or *Rspo2* single-gene mutant embryos. The expression of *Axin2*, a bona fide WNT/β-catenin signaling target gene, was severely downregulated in both the MxBA1/NPs and MdBA1 of DKO embryos compared to that in single *Wnt9b* or *Rspo2* mutants (Figure 2B). Furthermore, WNT9b and RSPO2 co-treatment in cultured whole BA1 explants induced *Axin2* expression in a cooperative manner (Figure 2C). Similarly, *Axin2* expression was induced cooperatively by WNT9b and RSPO2 in BA1 mesenchyme explants. RSPO2 alone could not induce *Axin2* expression in the BA1 mesenchyme explants because ectoderm-derived WNT9b (or other WNTs) is not present. Taken together, these results confirm cooperation between WNT9b and RSPO2 in the regulation of WNT/β-catenin signaling in developing facial structure.

### Cooperative Function by WNT9b and RSPO2 Regulates Gene Expression Within Facial Processes

**FIGURE 3:**
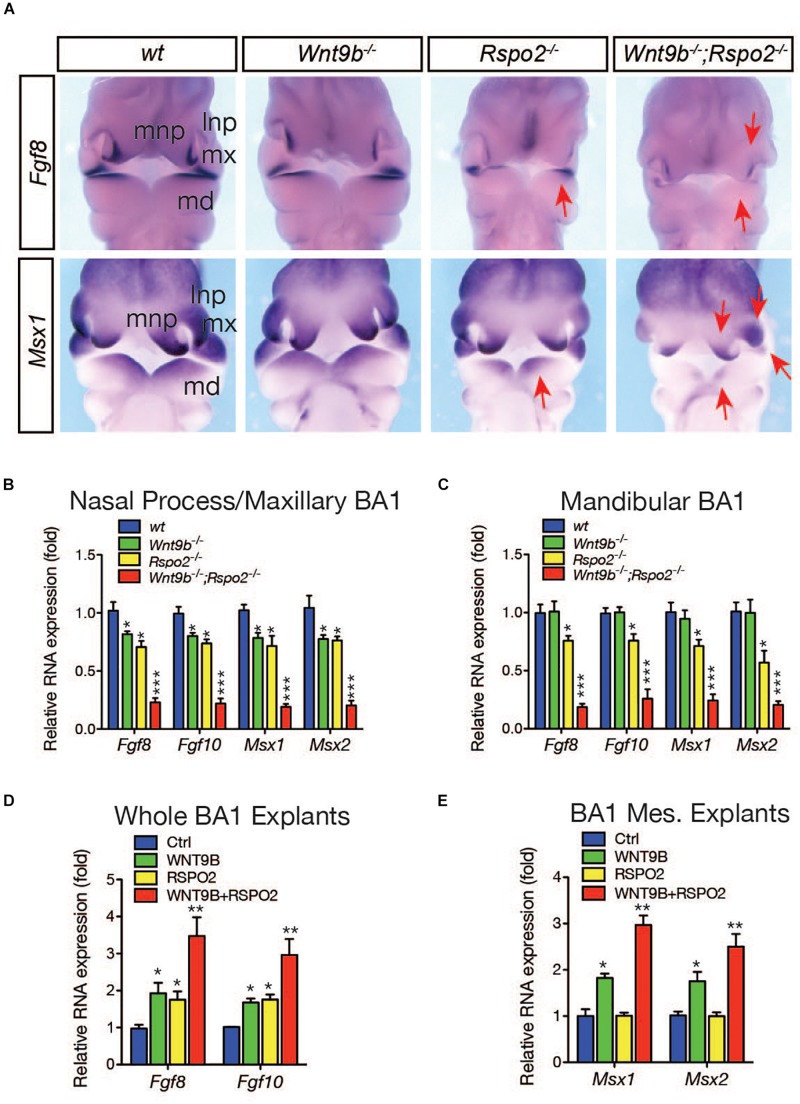
**(A)** Whole mount *in situ* hybridization analysis for *Fgf8* and *Msx1* gene expressions in wild-type, *Wnt9b*KO, *Rspo2*KO, and DKO embryos (*n* = 3) at E10.5. Red arrows indicate the reduced gene expression. **(B,C)** qRT-PCR analysis of marker gene expression in the facial process explants (*n* = 4) dissected from *Wnt9b*KO, *Rspo2*KO, and DKO embryos at E10.5. **(D,E)** qRT-PCR analysis of marker gene expression in the facial process explants (*n* = 4) cultured in the presence of WNT9b (20 ng/ml) and/or RSPO2 (200 ng/ml) proteins. *p < 0.05, **p < 0.01, and ***p < 0.005.

To determine whether cooperation between the RSPO2 and WNT9b is a major regulatory mechanism within facial processes, we examined the expression of *Fgf8* and *Msx1*, two WNT target genes expressed in the ectoderm and mesenchyme, respectively (Song et al., 2009; Wang et al., 2011), in DKO embryos. Expression of both genes was dramatically reduced in DKO embryos, indicating a loss of synergy between the *Wnt9b* and *Rspo2* genes (Figure 3A). In addition to *Fgf8* and *Msx1*, the expressions of additional markers, *Fgf10* and *Msx2*, were synergistically reduced in the upper facial processes and MdBA1 of DKO embryos (Figures 3B,C), providing strong evidence for cooperation between *Wnt9b* and *Rspo2* gene function.

Next, we examined the cooperative action of WNT9b and RSPO2 by utilizing *ex vivo* explant cultures. In a whole BA1 explant culture, the expression of ectodermal markers *Fgf8* and *Fgf10* was robustly induced by the co-treatment of WNT9b and RSPO2 at a level significantly higher than those induced by WNT9b or RSPO2 treatment alone (Figure 3D). In BA1 mesenchyme explants, co-treatment with WNT9b and RSPO2 generated even more evident cooperation between WNT9b and RSPO2. While RSPO2 was unable to induce the expression of mesenchymal markers *Msx1* and *Msx2* due to the absence of ectoderm-derived WNT, WNT9b, and RSPO2 together induced the expression of these marker genes to levels significantly higher than those with WNT9b treatment alone (Figure 3E).

**FIGURE 4:**
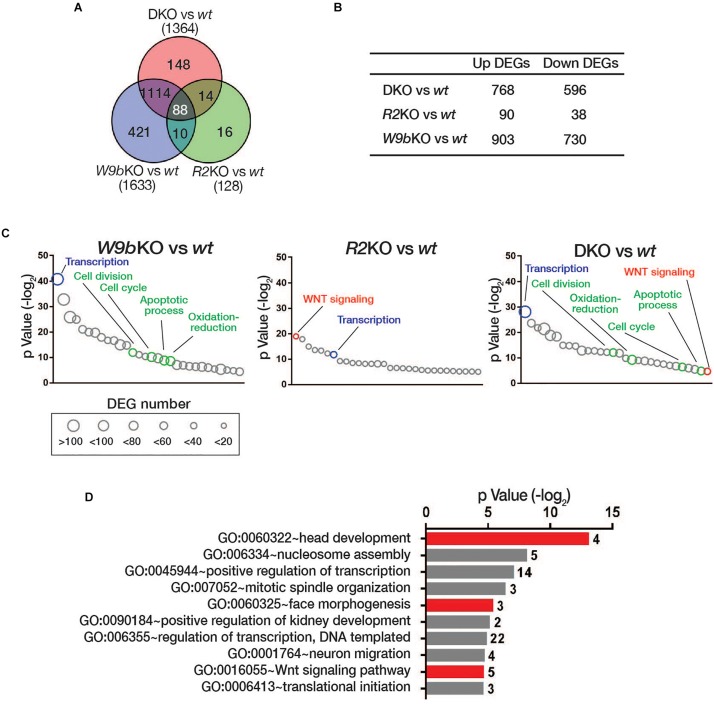
**(A)** Venn diagram of differential gene expression analysis in the facial processes of wild type, *Wnt9b*KO, *Rspo2*KO, and DKO by RNA sequencing. The numbers of the differentially expressed genes (DEGs) with more than 1.5-fold change in *Wnt9b*KO, *Rspo2*KO, and DKO compared to wild type (*n* = 3 for each genotype). **(B)** The number of DEGs upregulated or downregulated in *Wnt9b*KO, *Rspo2*KO, and DKO embryos. **(C)** Gene ontology analysis of the DEGs in *Wnt9b*KO, *Rspo2*KO, and DKO embryos, respectively. The biological processes that are common in all three KO backgrounds and are only associated with *Wnt9b*KO and DKO are shown in blue and green, respectively. Gene numbers in each GOTERM are represented by circles with different diameters. **(D)** Gene ontology analysis for the biological processes for the subgroup DEGs in DKO mice regulated by *Wnt9b:Rspo2* cooperative function. The number of genes belonging to the GOTERM are shown at the right end of each bar.

To determine the effect of the loss of the cooperative function by WNT9b and RSPO on global gene expression patterns during facial development, we sequenced RNA samples isolated from the facial primordia explants of *wild-type*, *Wnt9b*KO, *Rspo2*KO, and DKO embryos at E10.5, respectively. When compared with wild-type mice, 1,633, 128, and 1,364 differentially expressed genes (DEGs) that were either upregulated or downregulated by more than 1.5-fold were identified in *Wnt9b*KO, *Rspo2*KO, and DKO mice, respectively (Figures 4A,B). In the gene ontology analysis for the biological processes, the DEGs in *Wnt9b*KO did not show any significant association with WNT signaling (Figure 4C and Supplementary Table S1). This result may be explained in part by very weak *STF* reporter activity induced by WNT9b and relatively normal *TopGAL* expression in *Wnt9b* KO mice (Figures 1B,C). However, even though a relatively small number of DEGs were identified in *Rspo2* KO mice, we found that 10 DEGs in *Rspo2*KO mice are significantly associated with WNT signaling (Figure 4C and Supplementary Table S2). Interestingly, in DKO mice, 21 DEGs were found to be associated with WNT signaling, revealing an intensified association to WNT signaling compared to *Rspo2* KO or *Wnt9b* KO mice (Figure 4C and Supplementary Table S3).

We further selected 102 DEGs in DKO mice whose expression exhibited a synergistic pattern when compared to their expression levels in *Wnt9b* and *Rspo2* KO mice (Supplementary Figure S1). Gene ontology analysis of these DEGs also identified a significant association with WNT signaling (Figure 4D). Other notable biological processes identified were “regulation of transcription,” “face morphogenesis,” and “head development.” Taken together, both individual marker gene expressions and transcriptome analysis by RNA sequencing highlight the significance of WNT signaling cooperatively regulated by WNT9b and RSPO2. This process is crucial for the regulation of transcription within the facial processes and facial morphogenesis.

### Cellular and Morphological Defects Caused by Loss of WNT9B:RSPO2 Cooperation

**FIGURE 5:**
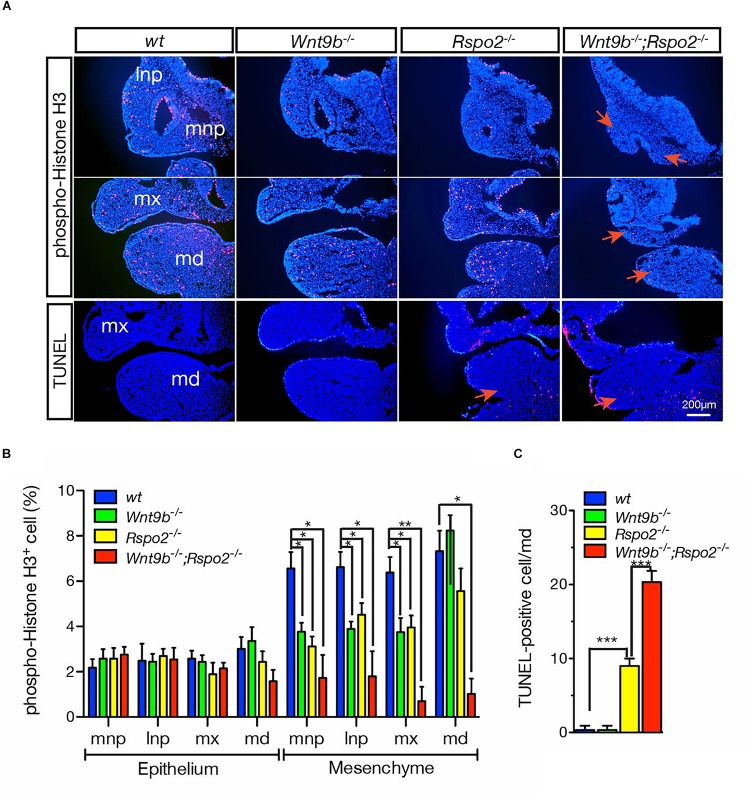
**(A)** Cell proliferation and apoptosis in the facial processes of wild-type, *Wnt9b*KO, *Rspo2*KO, and DKO embryos at E10.5. Immunofluorescence staining of phospho-histone H3 for cell proliferation and TUNEL staining for cell apoptosis were performed on cryosections. DAPI (blue) was used for nuclei counterstaining. Red arrows point at the area showing changes in cell proliferation and apoptosis. Three embryos of each genotype were analyzed. **(B)** Quantitation of phospho-histone H3-positive cells presented as a percentage of total cells. Three embryos for each mutant background were used for collecting the data. **(C)** Quantitation of TUNEL-positive cells presented as a percentage of total cells. Three embryos for each mutant background were used for collecting the data. Error bars represent the standard error of the mean (SEM). *p < 0.05; **p < 0.01; ***p < 0.005.

Our previous studies have demonstrated that cellular defects observed in *Rspo2* KO and *Wnt9b* KO mice are different (Jin et al., 2011, 2012). Cell proliferation evaluated by phospho-histone H3 expression was normal in the mesenchymal cells within the MdBA1 of both *Rspo2^–/–^* and *Wnt9b^–/–^* embryos (Figures 5A,B), consistent with our earlier results (Jin et al., 2011, 2012). However, there was a dramatic reduction in the number of proliferating cells within the mesenchymal compartment of the MdBA1 in DKO embryos at E10.5 (Figures 5A,B). These results clearly demonstrate that cooperation between WNT9b and RSPO2 is important for the regulation of cell proliferation. Because only significant loss of WNT signaling activity in DKO embryos results in cell proliferation defects, a mild or moderate level of WNT signaling in *Wnt9b^–/–^* or *Rspo2^–/–^* appears to be enough for the proliferation of the MdBA1 mesenchymal cells. In addition, cell proliferation within the MxBA1 and NP also significantly decreased in DKO embryos (Figures 5A,B). We previously demonstrated that the reduced cell proliferation in the MxBA1/NP of *Wnt9b^–/–^* embryos is dependent on FGF signaling activated by ectoderm-derived FGF8 and FGF10 (Jin et al., 2012). Because both *Fgf8* and *Fgf10* expression showed a synergistic reduction in the MdBA1 and MxBA1/NP of DKO mice (Figures 3A,B), WNT9b:RSPO2 cooperation likely regulates cell proliferation through FGF signaling in the facial processes.

We further found that WNT9b:RSPO2 cooperation also regulates cell death in the MdBA1. No significant cell death was evident in the MdBA1 of *Wnt9b^–/–^* embryos, whereas a significant amount of apoptotic cell death was observed within the aboral domain of MdBA1 in *Rspo2^–/–^* embryos (Figures 5A,C; Jin et al., 2011, 2012). In contrast, a synergistic increase in apoptotic cells was only observed within the mesenchymal compartment of the MdBA1 in DKO embryos at E10.5 (Figures 5A,C). These results suggest that WNT9b:RSPO2 cooperation is critical for cell survival in the mesenchymal compartment of the MdBA1. It appears that *TopGAL* expression levels are correlated with the extent of cell apoptosis. *TopGAL* expression in the MdBA1 of *Wnt9b^–/–^* embryos is very similar to that of wild-type embryos (Figure 2A). In contrast, *TopGAL* expression is significantly reduced in the MdBA1 of *Rspo2^–/–^* embryos and further reduced in DKO embryos. Furthermore, we previously showed that expression of *Fgf8*, *Edn1*, and *Bmp4*, WNT/β-catenin targets, is significantly reduced in the MdBA1 of *Rspo2^–/–^* embryos and proposed that disruption of multiple signaling activities may lead to cell apoptosis (Jin et al., 2011). Because severely reduced *Fgf8* and *Fgf10* expressions were detected in DKO embryos (Figures 3A,B), we assume that severe loss of WNT/β-catenin signaling activity may disrupt multiple signaling activities including FGF signaling and cause cell apoptosis.

Collectively, these results clearly demonstrate that WNT9B:RSPO2 cooperation is critical for both the proliferation and the survival of MdBA1 mesenchymal cells, whereas this cooperation may be less critical for cell proliferation in MxBA1 and NP mesenchymal cells. Furthermore, the identification through gene ontology analysis of a significant association of the DEGs in DKO with gene ontology terms such as “cell cycle,” “cell division,” and “apoptotic process” (Figure 4D) is supportive to these results.

**FIGURE 6:**
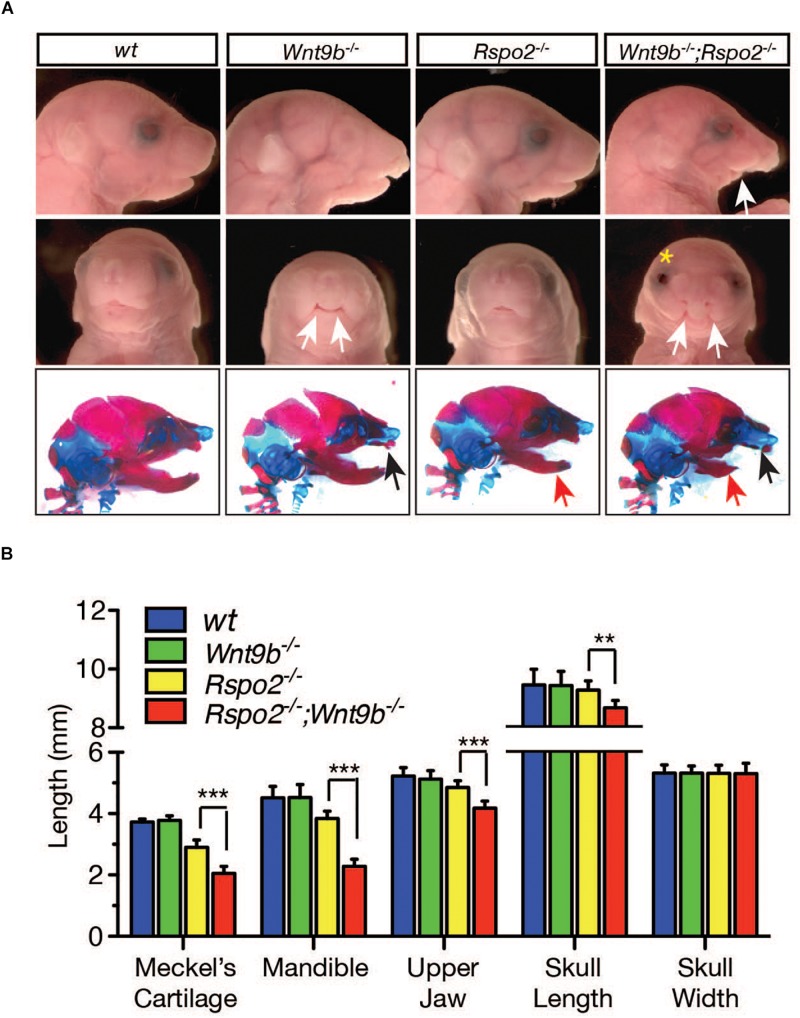
**(A)** Gross head morphology and skeleton (bottom panel) of wild-type, *Wnt9b*KO, *Rspo2*KO, and DKO animals at E18.5 obtained from mating between compound *Wnt9b^±^;Rspo2^±^* male and female mice. The compound *Wnt9b^±^;Rspo2^±^* mice were normal and fertile. Lateral view (top and bottom panels) and frontal view (middle panel) are shown. The white eye color of *Wnt9b*KO is not a specific phenotype but is due to the mixed genetic background of *Wnt9b*KO mice. White arrows indicate severe defect in the lower jaw (top panel) and cleft lip phenotype in the upper jaw (middle panel). Yellow asterisk indicates the open-eye phenotype observed in DKO mice. Black and red arrows in the bottom panel specify maxillary and mandibular defects, respectively. **(B)** Measurement of various parameters of skull dimensions. Three to four fetuses were used for the measurement. Error bars represent the standard error of the mean (SEM). **p < 0.01; ***p < 0.005.

To further determine how the loss of cooperation by RSPO2 and WNT9b affects facial structure development, we performed phenotypic analyses on *Wnt9b^–/–^* KO, *Rspo2^–/–^* KO, and *Wnt9b^–/–^;Rspo2^–/–^* DKO mice at E18.5. DKO mice exhibit significantly more severe facial defects than do either *Rspo2^–/–^* or *Wnt9b^–/–^* mice at E18.5 (Figure 6A). Mandibles of DKO mice were significantly smaller than those of *Rspo2^–/–^* or *Wnt9b^–/–^* mice. The cleft lip phenotype in DKO mice was more severe than the one detected in *Wnt9b^–/–^* mice. Of note, bilateral cleft lip, a phenotype never observed in *Rspo2^–/–^* mice, was observed in some *Rspo2^–/–^* mice in a *Wnt9b*^±^ background (50%, 3/6). In contrast, *Wnt9b^–/–^* mice in a *Rspo2*^±^ background showed the phenotype identical to *Wnt9b^–/–^* in a wild-type background. In addition, DKO mice exhibit some defects that were neither evident nor present in either *Rspo2^–/–^* or *Wnt9b^–/–^* mice. Upper jaw hypoplasia and open eyelids were never detected in *Wnt9b* or *Rspo2* single-mutant mice (Figure 6A). Measurements of skull dimensions and landmarks indicated that the lengths of the Meckel’s cartilage, mandible, upper jaw, and skull of DKO mice were significantly shorter than those of *Rspo2^–/–^* and *Wnt9b^–/–^* mice (Figure 6B), whereas the width of the skull was unchanged. Taken together, our results clearly show that while there are unique and independent functions of *Wnt9b* and *Rspo2* in facial development, a cooperative function between the *Wnt9b* and *Rspo2* genes plays a critical role in normal facial development and that a disruption of this cooperation results in severe cellular and morphological defects.

### A Possible Role of the LGR4 Family of Receptors in WNT9B:RSPO2 Cooperation During Facial Development

**FIGURE 7:**
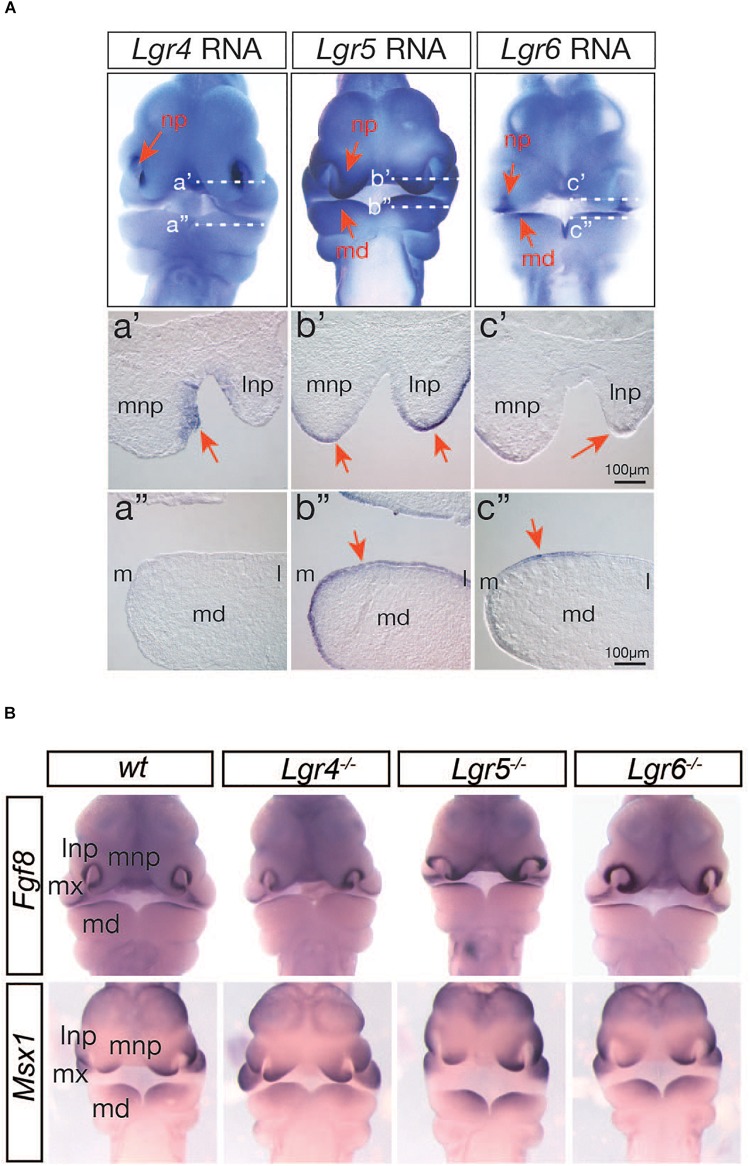
**(A)** Expression of *Lgr4/5/6* genes in the facial processes in mouse embryos at E10.5 determined by whole-mount *in situ* hybridization. Red arrows indicate lateral and medial nasal processes (np) and mandibular branchial arch 1 (md). Transverse section images of the matching stained embryos are presented. White-dotted lines (a’ and a”) indicated the section planes. Orientation of sections is indicated by lateral and medial nasal processes (lnp and mnp) and medial (m) and lateral (l) sides of the md. Only the right side of the md is presented. **(B)** Whole-mount *in situ* hybridization analysis of *Fgf8* and *Msx1* expression in *Lrg4*, *Lgr5*, and *Lgr6* KO mice (*n* = 4) at E10.5.

Three members of the LGR4 family of G protein-coupled receptors (LGR4, LGR5, and LGR6) play a central role as RSPO receptors for WNT:RSPO cooperation in many cellular contexts (Han et al., 2014; Krausova and Korinek, 2014; Gong et al., 2015; Zhang et al., 2017; Kriz and Korinek, 2018). However, it is still unclear whether the LGR4 family of receptors plays a central role in WNT9b:RSPO2 cooperation during embryonic facial development. Firstly, we assessed the expression of all three members of the *Lgr4* gene family within the developing facial structures of mouse embryos by whole-mount *in situ* hybridization and cryo-sectioning of the stained embryos. All of the *Lgr4*-family genes were expressed within the facial processes with a very distinctive but largely non-overlapping pattern at E10.5 (Figure 7A), suggesting that all LGR4-family receptors may play a key role in WNT9b:RSPO2 cooperation. Surprisingly, the expressions of *Fgf8* and *Msx1* genes were primarily unaffected within the facial process of *Lgr4*, *Lgr5*, and *Lgr6* gene KO embryos at E10.5 (Figure 7B). This suggests that the individual LGR4 family members do not have any function in *Fgf8* and *Msx1* expression.

**FIGURE 8:**
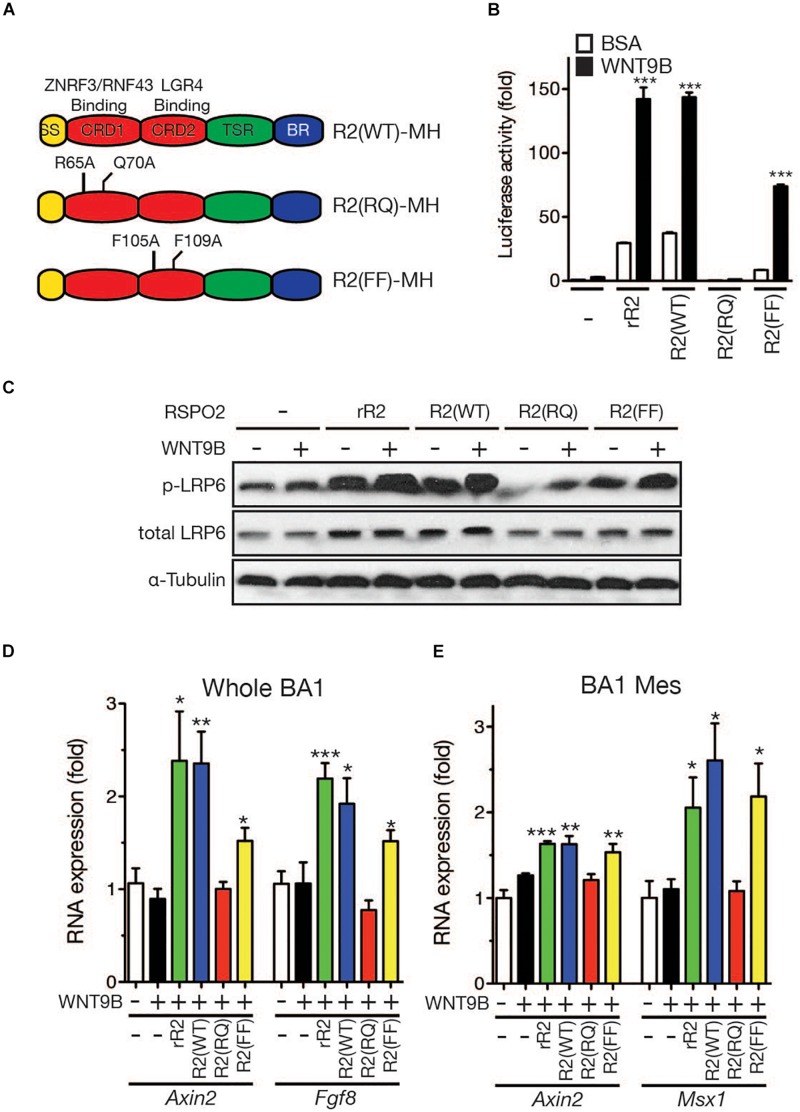
**(A)** Schematic structure of the RSPO2 protein and its derivatives. Mutated amino acid residues are labeled. **(B)** *STF* reporter assay to determine the cooperative activity between the WNT9b and RSPO2 mutant proteins. HEK293T cells were transfected, and reporter assay was performed as described in the Figure 1 caption. **(C)** LRP6 phosphorylation by Western blot analysis. Total LRP6 and α-tubulin expressions were also determined for normalization and loading controls. **(D,E)** qRT-PCR analysis of marker gene expression in the facial process explants (*n* = 4, whole BA1, whole mandibular branchial arch 1; BA1 Mes, mesenchymal part of the mandibular branchial arch 1) cultured in the presence of WNT9b (20 ng/ml) and/or RSPO2 (200 ng/ml) proteins. Error bars represent the standard error of the mean. *p < 0.05; **p < 0.01; ***p < 0.005.

Structural analysis of RSPO and LGR4 by X-ray crystallography previously identified key amino acids within the RSPO proteins that are required for the interactions with their receptors (Wang et al., 2013; Xu et al., 2013; Zebisch and Jones, 2015). In RSPO2, R65 and Q70 are required for binding ZNRF3/RNF43, whereas F105 and F109 are essential for binding the LGR4 family of receptors (Figure 8A). We produced recombinant RSPO2 proteins carrying mutations on those amino acids to determine whether RSPO2 binding to the LGR4 family is important for WNT9B:RSPO2 cooperation. The R2(RQ) mutant, in which both R65 and Q70 were converted to A, completely lost its ability to generate cooperation with WNT9B in the *STF* reporter assay (Figure 8B) and LRP6 phosphorylation in HEK293T cells (Figure 8C). Interestingly, the R2(FF) mutant that cannot bind the LGR4 family of receptors presented a significant cooperative activity with WNT9B, ∼50% of wild-type RSPO2 proteins (Figures 8B,C).

Next, we determined the cooperative activities of these RSPO2 mutant proteins in facial process explant culture. In whole BA1 explants, while co-treatment with wild-type RSPO2 and WNT9B effectively induced *Axin2* and *Fgf8* expression, co-treatment with WNT9B and R2(RQ) mutant protein failed to induce both marker genes (Figure 8D). Consistent with the results from HEK293T cells, the R2(FF) mutant, which is unable to bind LGR, significantly induced marker genes at 60-70% the level of wild-type RSPO2. In BA1 mesenchymal explants, the R2(FF) mutant protein exhibited a cooperative activity with WNT9B comparable to wild-type RSPO2 protein, while the R2(RQ) mutant was unable to generate any cooperative activity (Figure 8E). Together with the expression pattern of *Fgf8* and *Msx1* genes in *Lgr4* gene family mutant mice, these results suggest that the LGR4 family of receptors may not play a major role for RSPO2:WNT9B cooperation during facial development.

## Discussion

### Cooperative Activation of Canonical WNT Signaling by WNT9b and RSPO2 During Facial Morphogenesis in Mice

Canonical WNT signaling is one of the major signaling pathways that regulate facial morphogenesis during development. Disruption of canonical WNT signaling by gene knockout in mice or inherited mutations in humans clearly show the importance of the canonical WNT signaling pathway during facial morphogenesis (Niemann et al., 2004; Menezes et al., 2010; Jin et al., 2012; Fontoura et al., 2015). The RSPO family of proteins is a co-activator for canonical WNT signaling, generating a synergistic activation of canonical WNT signaling together with WNT ligands. Therefore, co-presence or single presence of the WNT and RSPO proteins will produce a range of WNT signaling activity, providing a fine-tuning of canonical WNT signaling.

Our study showed that the ectoderm-derived WNT9b ligand and the underlying mesenchyme-derived RSPO2 protein work together cooperatively to control canonical WNT signaling activity within the facial processes. Ablation of both the *Wnt9b* and *Rspo2* genes resulted in severe abnormalities in facial structures, whereas an individual loss of the *Wnt9b* or *Rspo2* gene showed milder or non-identical phenotypes. Transcriptome analysis showed that the genes associated with canonical WNT signaling were significantly affected in the *Rspo2* and *Rspo2;Wnt9b* double mutants, which is not the case in *Wnt9b* mutant mice. Therefore, cooperation between WNT9b and RSPO2 generates a different developmental outcome compared to WNT9b- or RSPO2-dependent outcomes. It is possible that the phenotypes detected in *Wnt9b* mutants may not be entirely the result of the disruption of canonical WNT signaling. Indeed, the *Wnt9b* gene function during kidney development has previously been shown to engage in both canonical and non-canonical WNT signaling (Carroll et al., 2005; Karner et al., 2009; Karner et al., 2011). Thus, some defects may be the result of a disruption of non-canonical WNT signaling. Furthermore, it is possible that some of the different outcomes may be partly a result of a differential strength of canonical WNT signaling, especially compared with the phenotypes in *Rspo2* and double mutants. While we did not investigate this in our study, an open-eye phenotype is only observed in double mutant mice, suggesting that strong canonical WNT signaling may be a key for proper eyelid development.

Within the facial processes, the *Wnt9b* gene is not the only *Wnt* gene known to be expressed (Summerhurst et al., 2008). The remaining question is whether WNT9b is the only WNT ligand working with RSPO2 or whether other expressed WNT ligands also cooperate with RSPO2 to regulate canonical WNT signaling within more specific parts of the facial structures. So far, no other *Wnt* genes, except *Wnt3*, have been linked to facial development. Humans carrying *WNT3* gene mutation display cleft lip/palate phenotype (Niemann et al., 2004). Therefore, WNT3 ligands are a potential candidate to cooperate with RSPO2. Since mice lacking the *Wnt3* gene die during gastrulation (Barrow et al., 2007), it is still unknown whether WNT3 is directly involved in facial development or not. Phenotypic analysis of compound mice heterozygous to the *Wnt3* gene and homozygous to the *Rspo2* gene would be interesting and provide a more complete picture of how canonical WNT signaling operates during facial development in mice.

### The Role of the LGR4 Family of Receptors in the Potentiation of WNT9b Signaling by RSPO2

The members of the LGR4 family of receptors are essential components for the regulation of RSPO’s cooperation with WNT ligands (Raslan and Yoon, 2019). They are involved in either inhibition of ZNRF3/RNF43 function or direct activation of canonical WNT signaling. It is obviously important to determine whether the LGR4 family of receptors are a part of WNT9b:RSPO2 cooperation during facial development. However, in this study, we did not observe any defects in facial morphology and marker gene expression in mice lacking each *Lgr4-*family gene. Because the LGR4-family receptors are known to have a similar function in RSPO binding and WNT signaling potentiation, our results suggest the possibility of either gene compensation or no functional role of the LGR4-family receptors. A largely non-overlapping expression pattern of the *Lgr4*-family genes in the facial structures may work against gene compensation. However, because the expression of genes of other *Lgr* families in each *Lgr4* family mutant is unknown, a possibility of gene compensation still remains. Interestingly, the facial phenotypes of *Lrg5;Lgr6* (Supplementary Figure S2), and *Lgr4;Lgr5* gene double-knockout mice (Kinzel et al., 2014) are relatively normal and did not mimic that of *Wnt9b;Rspo2* gene DKO mice, indicating that there is no compensation of gene functions between *Lgr5* and *Lgr6* or *Lgr4* and *Lgr6*.

A recent *in vitro* study strongly demonstrated that RSPOs, especially RSPO2 and RSPO3, potentiate WNT signaling independent of the LGR4-family receptors (Lebensohn and Rohatgi, 2018; Park et al., 2018; Szenker-Ravi et al., 2018). Most recently, *Lgr4;Lgr5;Lgr6* gene triple-knockout mice have been generated and their phenotypes described (Szenker-Ravi et al., 2018). In this study, *Rspo2* is shown to function independently of the *Lgr4* receptor family genes during limb development. Interestingly, the facial phenotype of *Lgr4;Lgr5;Lgr6* gene triple-knockout newborn mice did not show significant similarities to that of *Wnt9b;Rspo2* DKO except cleft palate. The expressions of facial prominence-specific WNT signaling target genes, such as *Fgf8* and *Msx1*, in the triple-knockout mice is currently not known and need to be examined in the future. An overall phenotype of triple-knockout mice and our collective data suggest that the LGR4-family receptors may play a less critical role in WNT9b:RSPO2 cooperation during facial development.

In conclusion, our data demonstrate that the RSPO2 protein potentiates canonical WNT9b-induced β-catenin signaling *in vivo* during mouse facial development. Interestingly, and consistent with other recent findings (Lebensohn and Rohatgi, 2018; Szenker-Ravi et al., 2018), WNT/β-catenin signaling potentiation by RSPO2 may be less dependent on the LGR4-family receptors in facial development. Our findings will have broad implications for WNT:RSPO cooperation, and they add to our understanding of normal physiological and pathological development associated with WNT signaling.

## Materials and Methods

### Animals

*Rspo2* null, *Rspo2^Δ*ZN*^* (an *Rspo2* null allele that a *LacZ* and neomycin-resistance gene cassette was removed), and *Wnt9b* mutant mice (Nam et al., 2007; Jin et al., 2011, 2012), and *Lgr4* mutant mice (Kato et al., 2007) were maintained in our laboratory. *Lgr5*, *Lgr6* mutant, and *TopGAL* mice were acquired from The Jackson Laboratory (Bar Harbor, ME, United States). The genotyping of these mice was performed as described previously (Jin et al., 2011, 2012) or according to the protocol available from The Jackson Laboratory. Mice were housed in a specific pathogen-free animal facility, and animal handling and experimental procedures were approved by the Institutional Animal Care and Use Committee of Maine Medical Center and Soonchunhyang University.

### Skeletal Preparation, β-Galactosidase Staining, and Whole-Mount *in situ* Hybridization

Skeletal preparation, whole-mount β-Galactosidase staining using X-gal substrate, and whole-mount *in situ* hybridization were performed as described in an earlier study (Jin et al., 2012). The fetuses and embryos were photographed under a Stemi SV6 stereomicroscope (Zeiss, Germany) and an Axioskop microscope (Zeiss, Germany) equipped with an AxioCam digital camera (Zeiss, Germany).

### Cell Proliferation and Apoptosis Assays

To determine apoptotic cell death, a TdT-mediated dUTP nick-end labeling (TUNEL) assay was carried out on 10 μm cryosections using an *in situ* cell death detection kit (Roche Applied Science, Penzberg, Germany) according to the manufacturer’s instructions. To evaluate cell proliferation, immunofluorescent staining with anti-phospho-histone H3 (1:100 dilution, Cell Signaling Technology, Danvers, MA, United States) was performed on cryosections. The nuclei positive for TUNEL or phospho-histone H3 were counted, and the percentage of total nuclei was calculated.

### Facial Process Explant Culture

For facial process explant culture, the facial primordial tissues were dissected from E10.5 embryos in cold phosphate-buffered saline (PBS). When necessary, the ectodermal layer was removed by incubating the explants with Dispase II (2.4 Unit/ml, Roche Applied Science, Penzberg, Germany) for 30 min. Isolated tissues were cultured for 24–36 h on filters floating in the transwell plates (Corning Inc., Corning, NY, United States) containing α-MEM (Minimum essential medium, Corning Inc., Corning, NY, United States) with 0.1 μg/ml ascorbic acid (Sigma-Aldrich, St. Louis, MO, United States), 10% (v/v) fetal bovine serum (FBS, Hyclone, Pittsburg PA, United States), and 1% (v/v) streptomycin/penicillin (Invitrogen, Carlsbad, CA, United States).

### Cell Culture

Human embryonic kidney 293T (HEK 293T) cells were acquired from the American Type Culture Collection (ATCC, Manassas, VA, United States) and maintained in DMEM (Dulbecco’s modified Eagle’s medium, Corning, United States) containing 10% (v/v) FBS and 1% (v/v) penicillin-streptomycin under 5% CO_2_ at 37°C. Recombinant RSPO2 and WNT9B proteins were obtained from R&D Systems (Minneapolis, MN, United States) and treated at the concentrations of 200 and 20 ng/ml, respectively.

### Total RNA Isolation and qRT-PCR

Total RNA was isolated from freshly dissected and cultured facial primordial explants of mouse embryos using TRIZOL (Sigma-Aldrich, St. Louis, MO, United States), and cDNA was synthesized from 0.8 μg RNA using a Proscript cDNA synthesis kit (New England Biolab, Ipswitch, MA, United States). qRT-PCR was performed using cDNA (10 ng RNA equivalent) as described previously (Jin et al., 2012). The sequences of the PCR primers were also described previously (Jin et al., 2011, 2012).

### Luciferase Reporter Assay and Western Blot Analysis

TopFlash plasmid DNA transfection was performed using TransIt-LT1 reagent (Mirus Bio, Madison, WI, United States) according to the manufacturer’s instructions. Briefly, DNA-TransIT mix was incubated with cells for 16 h, and the transfected cells were harvested 24 h after transfection for use in the luciferase assay.

Protein lysates for Western blot analysis were prepared by using a RIPA [10 mM Tris-Cl, pH 7.2, 2 mM EDTA, 150 mM NaCl, 1% Non-idet P-40, 0.1% SDS, 50 mM NaF, 1% sodium deoxycholate, 1 mM PMSF, 1X protease inhibitor mixture set V (EMD Chemicals, Gibbstown, NJ, United States), 0.2 mM sodium vanadate] lysis buffer. Protein lysates (10 μg) were resolved on 8% polyacrylamide gel and transferred to PVDF membranes. The membranes were probed with various primary antibodies against phospho-LRP6 (1:1,000 dilution, Cell Signaling Technology), LRP6 (1:1,000 dilution, Santa Cruz Biotechnology, Santa Cruz, CA, United States), and α-tubulin (1:1,000 dilution, Santa Cruz Biotechnology). Species- and isotype-matching secondary antibodies conjugated with horseradish peroxidase (HRP) were incubated, and the HRP signal was developed using the Pierce Super Signal West Dura kit (Thermo Scientific/Pierce, Rockford, IL, United States). The signals were measured using Image J software, and each experiment was performed in duplicate.

### Preparation of Recombinant RSPO2 Protein

Various RSPO2 mutant constructs carrying specific amino acid mutations were generated using the QuikChange Mutagenesis Kit (Agilent Technology, Santa Clara, CA, United States) from the pCDNA3.1A-mRspo2MH plasmid template encoding the wild-type mouse *Rspo2* open-reading frame (ORF) tagged with Myc-His at the C-terminus. Within the FU-CRD1 domain of RSPO2, we specifically introduced two alanine mutations into R65 and Q70 residues, which have been reported in human patients diagnosed with inherited anonychia. This domain is required for the interaction with ZNRF3/RNF43 receptors (Hao et al., 2012; Peng et al., 2013; Zebisch et al., 2013; Khalil et al., 2017). Within the FU-CRD2 domain of RSPO2, we also introduced two alanine mutations to the phenylalanine residues at residues 105 and 109, which is required for the interaction with LGR4-family receptors (de Lau et al., 2011; Wang et al., 2013). Wild type and mutant forms of *Rspo2* DNA constructs were transfected into HEK 293T cells using TransIt-LT1 reagent, and conditioned media were collected and concentrated. The activity of wild-type RSPO2 protein was determined by comparing it to the activity of the commercial recombinant RSPO2 protein in *Super TopFlash* (*STF*) WNT reporter assay in HEK 293T cells. Concentrations of mutant forms of RSPO2 were normalized with wild-type RSPO2 by Western blot analysis using anti-Myc antibody.

### RNA Sequencing and Data Analysis

Facial process explants, including mandibular and maxillary branchial arch and nasal processes, from E10.5 embryos were dissected in cold PBS, and total RNA was extracted using TRIZOL reagent (Invitrogen). RNA sequencing was performed on three samples per genotype at the Genome Technology Access Center at Washington University (St. Louis, MO, United States), and data analysis was performed by our colleagues at the Israel National Center for Personalized Medicine at Weizmann Institute of Science and Macrogen Inc. (Seoul, South Korea). More than 25 million reads per sample were acquired from the single-ended 50 bp sequencing on the HiSeq 2500. Poly-A/T stretches and Illumina adapters were trimmed from the reads using Cutadapt, and resulting reads shorter than 30 bp were discarded. Reads were mapped to the *Mus Musculus* GRCm38 reference genome using STAR. Expression levels for each gene were quantified using HTSeq-Count. DEGs showing more than 1.5-fold change with *p* < 0.05 in *Wnt9b*KO, *Rspo2*KO, and DKO mice were identified using DESeq2. Analysis of gene ontology for the biological processes was performed with the database for annotation, visualization, and integration discovery (DAVID; Ver. 6.8) online tool.

### Statistical Analysis

Three to four embryos and fetuses for each genotype were used in most experiments unless otherwise stated. The experimental data were analyzed by non-paired Student’s *t*-test using GraphPad Prism software (GraphPad Software, La Jolla, CA, United States). Statistical significance was set at *p* < 0.05.

## Data Availability Statement

The datasets generated for this study can be found in NCBI GEO accession GSE147474.

## Ethics Statement

The animal study was reviewed and approved by Institutional Animal Care and Use Committee of Soonchunhyang University and Maine Medical Center.

## Author Contributions

Y-RJ: conceptualization, investigation, and writing-original draft. XH: investigation. KN: resource. DB-A: data analysis. YO: investigation and formatting. JS: conceptualization, writing – reviewing and editing, and supervision. JY: conceptualization, writing – reviewing and editing, supervision, and funding.

## Conflict of Interest

The authors declare that the research was conducted in the absence of any commercial or financial relationships that could be construed as a potential conflict of interest.
