# Supplementary material for: Canonical WNT/β-Catenin Signaling Activated by WNT9b and RSPO2 Cooperation Regulates Facial Morphogenesis in Mice
Source: Front Cell Dev Biol. 2020 May 8;8:264. doi: 10.3389/fcell.2020.00264 (PMC7225269; doi:10.3389/fcell.2020.00264)
Supplement: Supplementary file 1 [file Data_Sheet_1.pdf]

**Supplementary figure legends**

**Figure S1**

Expression profile of the subgroup DEGs in DKO mice regulated by cooperative function between the *Wnt9b* and *Rspo2* genes. Among 1364 DEGs in DKO background, genes that show the expression change is greater than the addition of changes in *Rspo2* KO and *Wnt9b* KO mice. 102 genes displaying synergistic changes are shown.

**Figure S2**

(A-D) Frontal face morphology of *Lgr5;Lgr6* compound KO. (E-H) H&E staining of the head sagittal sections of *Lgr5;Lgr6* compound KO mice at E18.5. Arrows indicate the fusion between tongue and lower jaw. Abbreviations: lj, lower jaw; ps, palatal shelf; t, tongue.
