# Supplementary figures and images for: Canonical WNT/β-Catenin Signaling Activated by WNT9b and RSPO2 Cooperation Regulates Facial Morphogenesis in Mice
Source: Front Cell Dev Biol. 2020 May 8;8:264. doi: 10.3389/fcell.2020.00264 (PMC7225269; doi:10.3389/fcell.2020.00264)

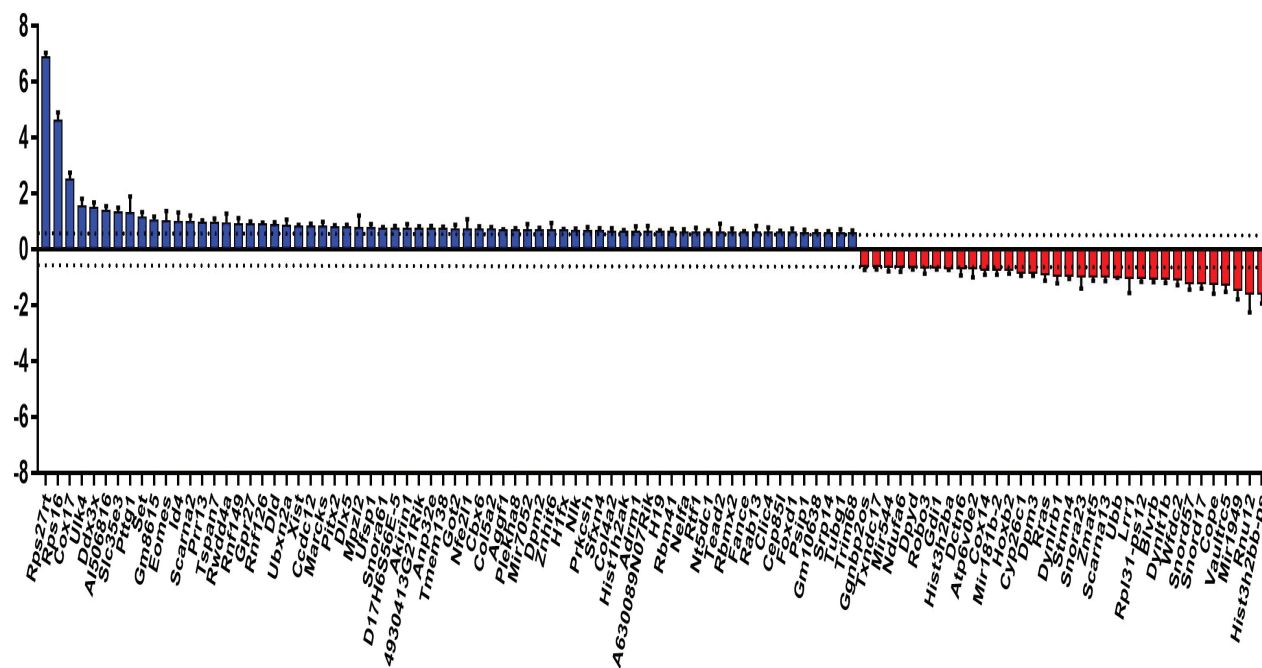

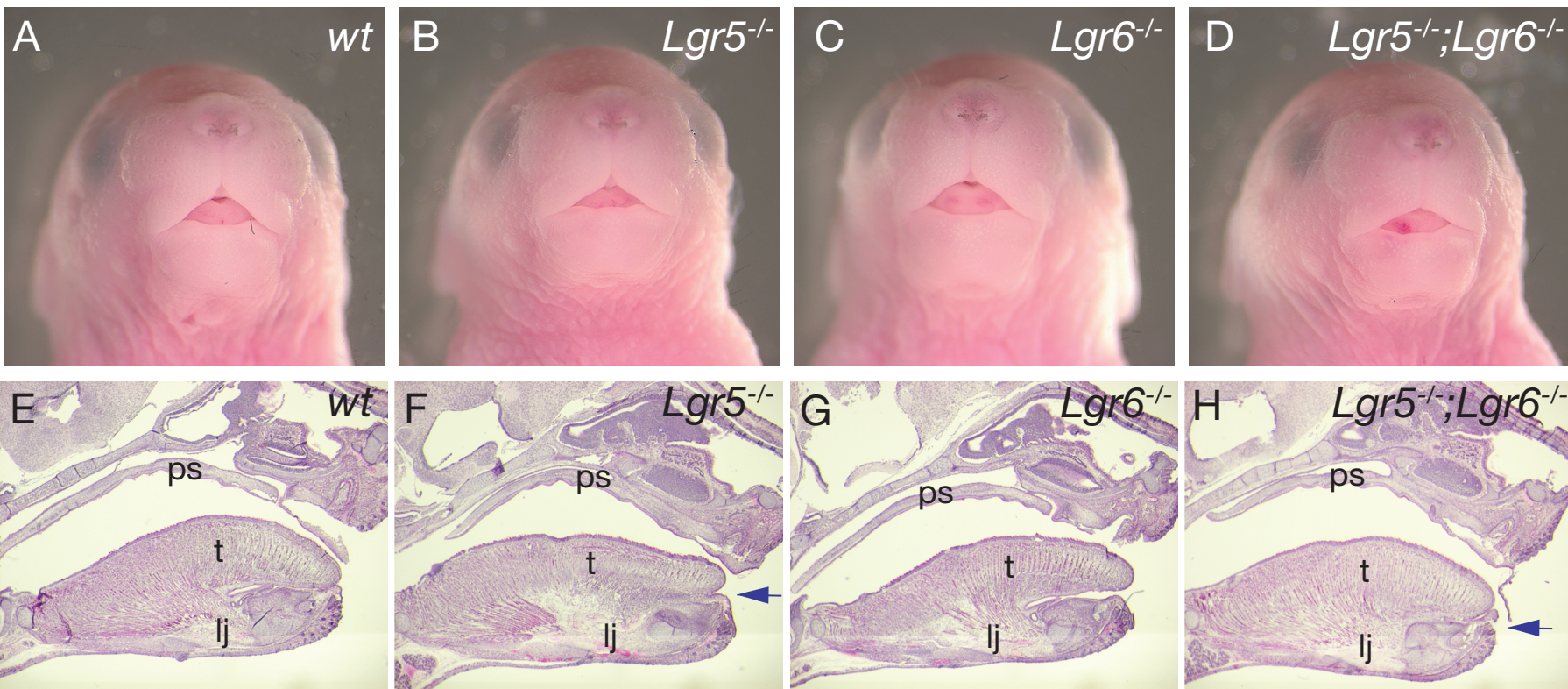

Supplement: Supplementary file 2 [file Image_1.pdf]
