# Supplementary material for: Canonical WNT/β-Catenin Signaling Activated by WNT9b and RSPO2 Cooperation Regulates Facial Morphogenesis in Mice
Source: Front Cell Dev Biol. 2020 May 8;8:264. doi: 10.3389/fcell.2020.00264 (PMC7225269; doi:10.3389/fcell.2020.00264)
Supplement: Supplementary file 3 [file Table_1.pdf]

Supplementary Table1. Gene Ontology_Biological Process (*W9b*KO vs *wt*)

**Process**	***p* value**	**Gene count**
GO:0006351~transcription, DNA-templated	5.13E-13	223
GO:0006355~regulation of transcription, DNA-templated	1.34E-10	248
GO:0045944~positive regulation of transcription from RNA polymerase II promoter	1.64E-08	123
GO:0006397~mRNA processing	3.09E-08	54
GO:0008380~RNA splicing	4.54E-07	42
GO:0016569~covalent chromatin modification	1.00E-06	44
GO:0045893~positive regulation of transcription, DNA-templated	1.14E-06	76
GO:0001701~in utero embryonic development	4.13E-06	46
GO:0007420~brain development	9.40E-06	36
GO:0007399~nervous system development	1.01E-05	53
GO:0000122~negative regulation of transcription from RNA polymerase II promoter	3.22E-05	85
GO:0007067~mitotic nuclear division	3.68E-05	41
GO:0051301~cell division	2.59E-04	48
GO:0007507~heart development	4.65E-04	36
GO:0098609~cell-cell adhesion	7.57E-04	28
GO:0007049~cell cycle	8.79E-04	68
GO:0043066~negative regulation of apoptotic process	0.001226	63
GO:0006915~apoptotic process	0.002311	62
GO:0055114~oxidation-reduction process	0.002636	71
GO:0045892~negative regulation of transcription, DNA-templated	0.007664	60
GO:0030335~positive regulation of cell migration	0.00847	26
GO:0007155~cell adhesion	0.011029	51

GO:0016567~protein ubiquitination	0.012096	40
GO:0006974~cellular response to DNA damage stimulus	0.012534	45
GO:0030154~cell differentiation	0.015857	75
GO:0006412~translation	0.022867	42
GO:0007275~multicellular organism development	0.024526	94
GO:0006281~DNA repair	0.028353	4
GO:0008283~cell proliferation	0.029314	5
GO:0015031~protein transport	0.030745	4

*Top 30 GO_BPs are listed

Supplementary Table2. Gene Ontology_Biological Process (*R2*KO vs *wt*)

**Process**	***p* value**	**Gene count**
GO:0016055~Wnt signaling pathway	1.97E-06	10
GO:0045944~positive regulation of transcription from RNA polymerase II promoter	4.26E-06	19
GO:0007399~nervous system development	3.16E-05	11
GO:0048625~myoblast fate commitment	8.34E-05	3
GO:0007275~multicellular organism development	9.67E-05	17
GO:0090090~negative regulation of canonical Wnt signaling pathway	2.11E-04	6
GO:0006351~transcription, DNA-templated	2.91E-04	23
GO:0000122~negative regulation of transcription from RNA polymerase II promoter	0.001658	12
GO:0030514~negative regulation of BMP signaling pathway	0.001866	4
GO:0030154~cell differentiation	0.002807	12
GO:0016569~covalent chromatin modification	0.002943	7
GO:0016477~cell migration	0.003511	6
GO:0045893~positive regulation of transcription, DNA-templated	0.003518	10
GO:0006355~regulation of transcription, DNA-templated	0.003575	23
GO:0043588~skin development	0.003623	4
GO:0030900~forebrain development	0.0108	4
GO:0010975~regulation of neuron projection development	0.011016	3
GO:0060070~canonical Wnt signaling pathway	0.011849	4
GO:0035116~embryonic hindlimb morphogenesis	0.013234	3
GO:0001843~neural tube closure	0.014925	4
GO:0060325~face morphogenesis	0.019087	3
GO:0001701~in utero embryonic development	0.021578	6

GO:0008584~male gonad development	0.021801	4
GO:0001649~osteoblast differentiation	0.022311	4
GO:0009791~post-embryonic development	0.022828	4
GO:0035019~somatic stem cell population maintenance	0.024813	3
GO:0001764~neuron migration	0.028353	4
GO:0030324~lung development	0.028353	4
GO:0007166~cell surface receptor signaling pathway	0.029314	5
GO:0048511~rhythmic process	0.030745	4

*Top 30 GO_BPs are listed

Supplementary Table 3. Gene Ontology_Biological Process (DKO vs *wt*)

**Process**	***p* value**	**Gene count**
GO:0006351~transcription, DNA-templated	3.37E-09	178
GO:0006397~mRNA processing	7.71E-08	47
GO:0006334~nucleosome assembly	2.74E-07	23
GO:0006355~regulation of transcription, DNA-templated	3.61E-07	197
GO:0045944~positive regulation of transcription from RNA polymerase II promoter	2.11E-06	99
GO:0045893~positive regulation of transcription, DNA-templated	2.96E-06	65
GO:0008380~RNA splicing	3.23E-05	33
GO:0098609~cell-cell adhesion	3.63E-05	28
GO:0007067~mitotic nuclear division	4.07E-05	36
GO:0000122~negative regulation of transcription from RNA polymerase II promoter	1.34E-04	71
GO:0006417~regulation of translation	1.43E-04	20
GO:0007420~brain development	1.49E-04	29
GO:0001701~in utero embryonic development	1.90E-04	36
GO:0016569~covalent chromatin modification	2.15E-04	33
GO:0051301~cell division	2.36E-04	42
GO:0007399~nervous system development	2.78E-04	42
GO:0030036~actin cytoskeleton organization	0.001116	20
GO:0055114~oxidation-reduction process	0.001668	62
GO:0007411~axon guidance	0.001976	20
GO:0006974~cellular response to DNA damage stimulus	0.00231	42
GO:0007507~heart development	0.002955	29
GO:0006281~DNA repair	0.004208	33

GO:0016567~protein ubiquitination	0.005397	36
GO:0008285~negative regulation of cell proliferation	0.007694	37
GO:0045892~negative regulation of transcription, DNA-templated	0.009501	51
GO:0007049~cell cycle	0.011864	53
GO:0007155~cell adhesion	0.01574	43
GO:0043066~negative regulation of apoptotic process	0.022301	48
GO:0006915~apoptotic process	0.035327	47
GO:0016055~Wnt signaling pathway	0.038978	21

*Top 30 GO_BPs are listed
